# Transcriptomics of shading-induced and NAA-induced abscission in apple (*Malus domestica*) reveals a shared pathway involving reduced photosynthesis, alterations in carbohydrate transport and signaling and hormone crosstalk

**DOI:** 10.1186/1471-2229-11-138

**Published:** 2011-10-17

**Authors:** Hong Zhu, Chris D Dardick, Eric P Beers, Ann M Callanhan, Rui Xia, Rongcai Yuan

**Affiliations:** 1 Alson H. Smith, Jr. Agricultural Research and Extension Center, Virginia Polytechnic Institute and State University, 595 Laurel Grove Road, Winchester, VA 22602, USA; 2 Appalachian Fruit Research Station, United States Department of Agriculture, Agricultural Research Service, Kearneysville, WV, 25430, USA; 3 Department of Horticulture, Virginia Polytechnic Institute and State University, Blacksburg, VA 24061, USA

## Abstract

**Background:**

Naphthaleneacetic acid (NAA), a synthetic auxin analogue, is widely used as an effective thinner in apple orchards. When applied shortly after fruit set, some fruit abscise leading to improved fruit size and quality. However, the thinning results of NAA are inconsistent and difficult to predict, sometimes leading to excess fruit drop or insufficient thinning which are costly to growers. This unpredictability reflects our incomplete understanding of the mode of action of NAA in promoting fruit abscission.

**Results:**

Here we compared NAA-induced fruit drop with that caused by shading via gene expression profiling performed on the fruit abscission zone (FAZ), sampled 1, 3, and 5 d after treatment. More than 700 genes with significant changes in transcript abundance were identified from NAA-treated FAZ. Combining results from both treatments, we found that genes associated with photosynthesis, cell cycle and membrane/cellular trafficking were downregulated. On the other hand, there was up-regulation of genes related to ABA, ethylene biosynthesis and signaling, cell wall degradation and programmed cell death. While the differentially expressed gene sets for NAA and shading treatments shared only 25% identity, NAA and shading showed substantial similarity with respect to the classes of genes identified. Specifically, photosynthesis, carbon utilization, ABA and ethylene pathways were affected in both NAA- and shading-induced young fruit abscission. Moreover, we found that NAA, similar to shading, directly interfered with leaf photosynthesis by repressing photosystem II (PSII) efficiency within 10 minutes of treatment, suggesting that NAA and shading induced some of the same early responses due to reduced photosynthesis, which concurred with changes in hormone signaling pathways and triggered fruit abscission.

**Conclusions:**

This study provides an extensive transcriptome study and a good platform for further investigation of possible regulatory genes involved in the induction of young fruit abscission in apple, which will enable us to better understand the mechanism of fruit thinning and facilitate the selection of potential chemicals for the thinning programs in apple.

## Background

Most apple trees tend to bear more fruit than they can support to maturity. While such over-cropping may help ensure reproductive success, it can lead to branch damage, low quality fruit and drastic reductions in cropping in the following year. Consequently, over-cropping is an undesirable trait. Although a self-thinning process known as the "June drop" can help alleviate the negative impact of excessive fruit bearing, apple growers often find it necessary to apply chemical thinners to remove excess fruit at an early stage of fruit development. Naphthaleneacetic acid (NAA) is one of the most commonly used chemical thinners, but its efficacy varies among different varieties and is affected by environmental conditions following the application.

The physiological mechanisms by which NAA promotes the abscission of young apple fruitlets have been discussed [1-3]. Principal among these mechanisms is a reduction in carbohydrate availability to the developing fruit either by interference with photosynthesis [4,5] or by reduced translocation of metabolites, including photosynthates, from leaves to the fruit [6]. The importance of photosynthesis and photosynthate translocation in fruit retention is further illustrated by experiments involving shading or removal of leaves, two treatments that cause extensive apple fruit abscission [7,8]. Moreover, normal fruitlet abscission, which can occur both shortly after anthesis and during the "June drop", has been at least partly attributed to the competition for carbohydrates among young fruit and between fruit and vegetative shoots [7,9]. Together these findings indicate that photosynthesis is critical for fruit development and treatments that alter the levels of carbohydrates available for translocation to the developing fruit can influence the fruit set of apple trees.

In addition to its effects on carbohydrate levels, NAA application apparently enhances apple fruitlet abscission through increased ethylene production [10-13]. Application of ethephon, which releases ethylene, effectively promoted the abscission of young fruit in apple [14], while aminoethoxyvinylglycine (AVG), a strong inhibitor of ethylene biosynthesis, reduced fruit ethylene production and young fruit abscission in apple [13,15]. The NAA-induced increase in ethylene production is positively correlated with changes in the expression of ethylene biosynthesis and signal transduction genes, including five ACC synthase genes (*MdACS*), one ACC oxidase gene (*MdACO*), four ethylene receptor genes (*MdETR *and *MdERS*) and one ethylene signal transduction gene (*MdCTR1*) [12,13,16]. It has been reported that apple fruitlet abscission is preceded by a stimulation of ethylene biosynthesis and an acquisition in the sensitivity to ethylene [12]. Also, a recent microarray analysis of the abscission-related transcriptome in tomato flower abscission zone (AZ) revealed a link between the acquisition of ethylene sensitivity in the AZ and altered expression of auxin-regulated genes due to auxin depletion [17].

Cell wall breakdown and cell separation are required within the fruit abscission zone (FAZ) for fruit abscission. Cell wall remodeling genes are induced in the FAZ [18,19] and the activities of cell wall remodeling and degrading enzymes, including expansin, pectate lyase, polygalacturonase and β-1,4-glucanase, have been shown to markedly increase, concomitant with increased ethylene production, catalyzing the loosening and breakdown of the cell wall and promoting fruit drop [20-23].

The findings summarized above suggest that abscission-associated carbohydrate-, ethylene- and auxin-responsive signaling pathways engage in crosstalk with each other and with other signaling pathways to coordinate abscission. Increasing knowledge of changes in gene expression associated with abscission will aid in the development of strategies for more predictable thinning results and set the stage for the development of improved thinners. Moreover, the identification of regulatory networks that are central to apple fruit abscission will enhance our basic understanding of organ abscission, which is a fundamental aspect of plant development. In this study, we compared NAA and shading-induced abscission, through transcriptome analysis, to reveal the molecular mechanism controlling the induction of apple fruitlet abscission. Results show that NAA, like shading, imposes a stress signal through photosynthesis impairment and causes altered hormone signaling and triggers fruit abscission.

## Results

### Effects of NAA and shading on young fruit abscission and ethylene production by young fruit and leaves

**Figure 1:**
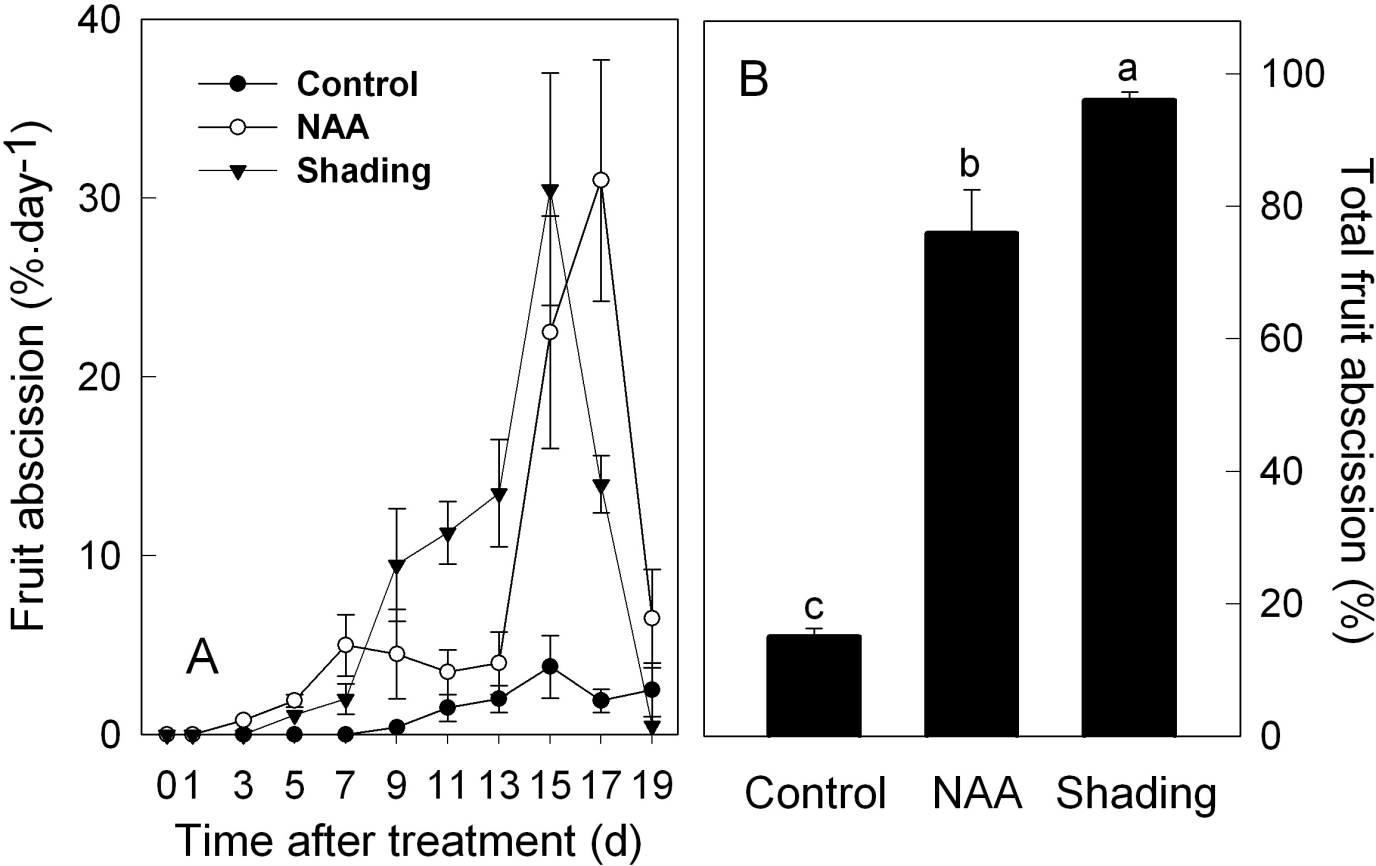
**Effect of NAA and shading treatments on fruit abscission of 'Golden Delicious' apples**. (A) NAA and shading increase fruit abscission rate. (B) NAA and shading treatment also increase the percentage of total fruit drop. Results represent the mean (± SE) of three replicates. Different letters indicate significant differences among means according to Duncan's multiple range test (*P *≤ 0.05).

A comparison of the relative effectiveness of NAA and shading as inducers of fruit abscission revealed significant treatment-specific differences in abscission rates and totals. For example, while both treatments promoted detectable increases in abscission rates within the first 7 d post-treatment, the NAA-induced abscission rate remained essentially unchanged from 7 to 13 d, whereas shading resulted in a relatively steady increase in the rate of fruit abscission for the same period (Figure 1A). By 15 d, however, similar rates of abscission were observed for both treatments and rates were near or below control rates by 19 d for both treatments. Ultimately, shading was significantly more effective in promoting fruit drop, causing 98% of the fruit to abscise within the 19-d period of the study, compared to a 75% loss in the same period following NAA treatment. Interestingly, the pattern of abscission exhibited by controls roughly mirrored that of treated trees but resulted in less than 10% of the fruit being shed, indicating that the NAA and shading treatments were able to act additively or synergistically with the endogenous early fruit abscission program (Figure 1B).

**Figure 2:**
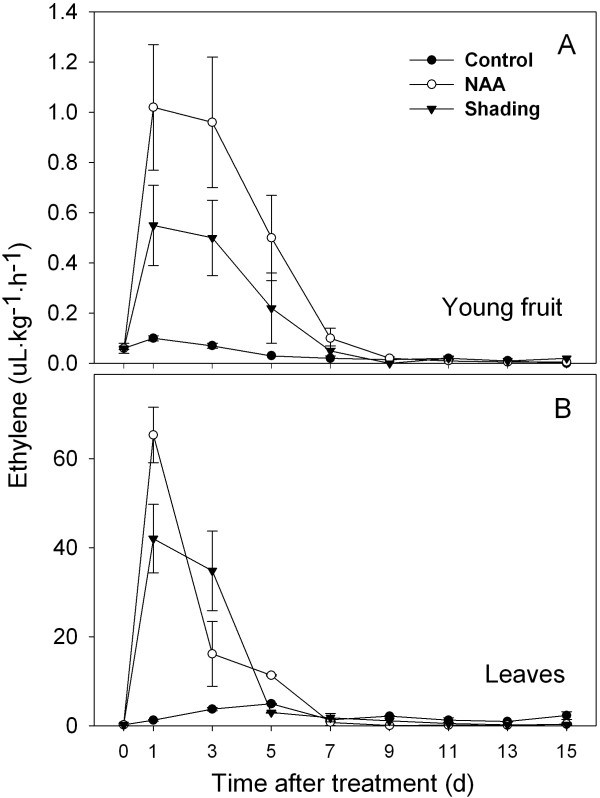
**Effect of NAA and shading treatments on ethylene production of 'Golden Delicious' apples**. (A) NAA and shading induce ethylene in young fruit. (B) NAA and shading also induce ethylene in leaves. Results represent the mean (± SE) of three replicates.

Previous studies have documented the link between NAA- and shading-induced abscission and ethylene production. We confirmed that the major increases in ethylene production preceded the onset of fruit abscission (Figure 2A compared with Figure 1A). The maximum level of NAA-induced fruit ethylene production was detected at 1 d and had decreased to control level by 7 d after treatment. Shaded fruit also released higher levels of ethylene relative to the control between 1 and 5 d, but levels reached only 50% of those from NAA-treated fruit (Figure 2A). A similar pattern of ethylene release was detected for leaves, where the ethylene production of both shaded and NAA-treated leaves peaked at 1 d, but decreased to control level by 5 and 7 d after treatment, respectively. However, at its peak rate, measured at 1 d, almost 70-fold more ethylene was released by leaves compared to fruit (Figure 2B).

### Expression profiling of young fruit abscission induced by NAA and shading

To identify genes whose expression patterns correlated with the fruit abscission induced by NAA and shading, gene expression profiling was performed using the FAZ at three time points (D1, D3 and D5), a period spanning the earliest phase of the treatment-dependent increase in abscission above control levels (Figure 1). For each time point, labeled cDNA from the FAZ of NAA- or shading-treated trees was hybridized to reference cDNA from the FAZ of non-treated trees for the same time point, so as not to confound the changes in gene expression caused by treatments with those occurring during fruit development.

Seven-hundred-twenty-two genes from NAA-treated sample hybridizations and 1057 genes from shading-treated sample hybridizations showed statistically significant changes in expression (Additional file 1, Table S1). Of these genes, 168 were differentially expressed in FAZs from both NAA- and shading-treated samples, and 86% (145) of those displayed similar expression patterns, indicating that NAA- and shading-induced abscission share some common signaling pathways.

Time points and selected genes were grouped according to expression pattern by hierarchical cluster analysis (Additional file 2, Figure S1). Following NAA treatment, the largest number of differentially expressed genes was detected at 3 d, followed by 5 and 1 d after NAA treatment, suggesting that NAA caused a transient effect during the abscission induction. In contrast, shading led to a sustained increase in the number of differentially expressed genes from 1 to 5 d. For both treatments, there were approximately equal numbers of genes showing upregulation or downregulation by 1 d, but induced genes outnumbered repressed genes for 3 and 5 d.

The two array datasets were further analyzed using K-means clustering (KMC) for genes whose expression pattern was correlated with the induction of fruit abscission (Additional file 3, Table S2). The cluster names were assigned upregulated (*u*), unchanged (*o*) or downregulated (*d*) for each time point. NAA-responsive genes were classified into 8 main clusters. As shown in Additional file 4 (Figure S2), the largest group (comprised of clusters 2 and 6) of differentially regulated genes was detected at 3 d. Shading-responsive genes were divided into 10 clusters. Similar to the NAA dataset, most clusters reflected either up- or down-regulation at one or two time points. In contrast to the NAA treatment, two clusters in the shading dataset (2 and 7) showed a persistent repression or induction at all three time points, and one cluster (5) comprised 51 genes that were first downregulated at 1 d, but later upregulated at 5 d after treatment (Additional file 4, Figure S2).

**Figure 3:**
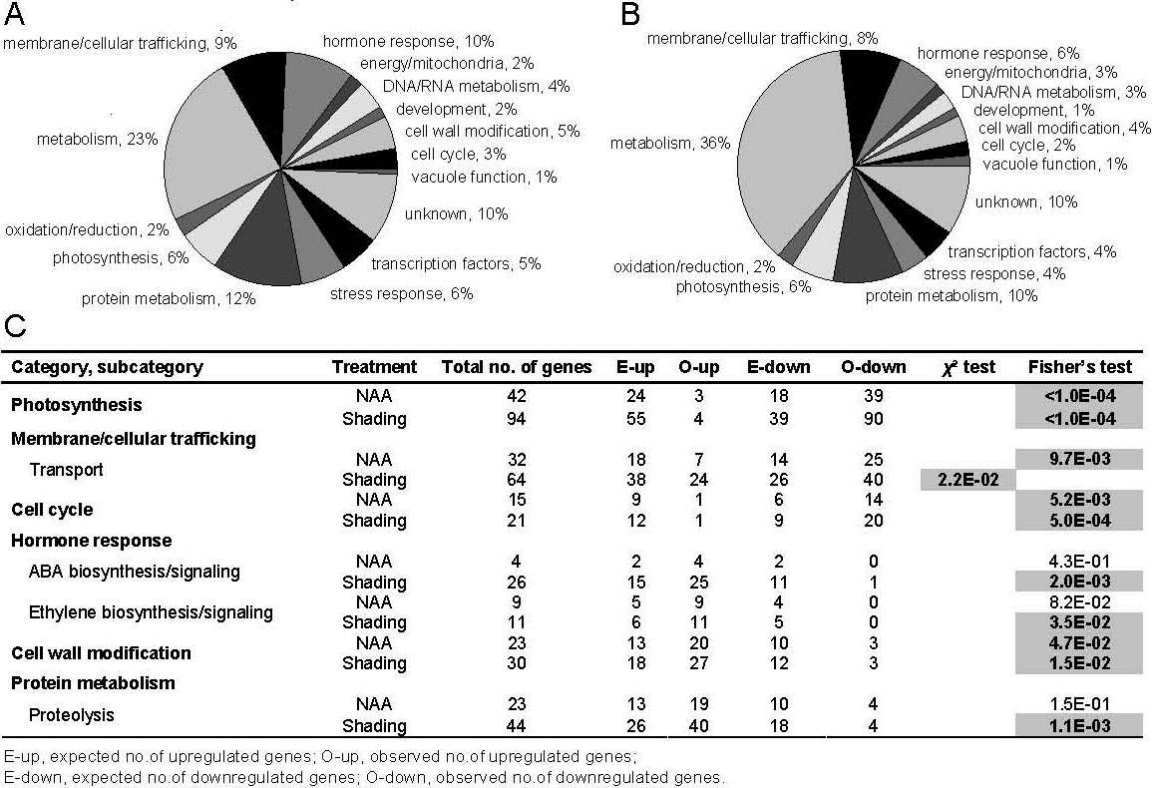
**Functional categories of statistically significant genes**. (A) Differentially expressed genes are categorized from NAA-treated FAZ. (B) Differentially expressed genes are categorized from shading-treated FAZ. The functional categorization is based on the annotation and GO information. Category names are indicated near each pie slice, along with the proportion of each category. (C) Functional categories showing non-random expression trends. Statistically significant values are highlighted (*P *≤ 0.05).

We examined gene expression patterns to identify functional categories correlated with fruit abscission. Genes probed by the apple array have both annotation and gene ontology (GO) information. However, as some annotations and GO categories do not provide detailed information on the biological mechanisms, additional manual annotations and literature validations were conducted for the entire list of differentially expressed genes. The resulting 15 functional categories included eight categories (photosynthesis, metabolism, membrane/cellular trafficking, cell cycle, hormone response, cell wall modification, protein metabolism and transcription factors) that accounted for over 70% of all the differentially expressed genes for both treatments (Figure 3A-B). After further gene categorization, we found that some categories and their subcategories showed trends where most members were either up- or downregulated by one or both treatments. To determine if such expression trends were statistically significant or just occurred by chance, χ^2 ^and Fisher's exact tests were performed on these categories and their subcategories, showing most, but not all categories and subcategories with non-random expression trends. A similar approach was used by Dardick et al (2007) for the enrichment analysis [24]. Those statistically significant categories and subcategories were shown in Figure 3C. All the resulting classifications were displayed in Additional file 5 (Table S3) and used for the subsequent analysis.

### Photosynthesis-related genes

The chloroplast is the site of the energy transduction and Calvin Cycle phases of photosynthesis and starch metabolism. Reductions in chloroplast function in response to shading have been reported previously [7] and were consistent with our transcript profiles from shading-treated trees. We found that NAA treatment also led to strong reductions in photosynthesis-related gene expression, which supported a previous report [4]. Although shading downregulated a larger number of photosynthesis-related genes than NAA, in both treatments over 90% of the differentially expressed genes related to chloroplast function were repressed (Additional file 5, Table S3). The affected genes function in light-harvesting, oxygen evolution, electron transport and carbon fixation. Shading-repressed genes were also involved with chlorophyll biosynthesis, chloroplast DNA binding, thylakoid formation and carbon utilization. However, only a small overlap was observed between this latter group of shading-repressed genes and those repressed by NAA, indicating that NAA and shading repress photosynthesis-related processes through partially distinct mechanisms.

### Carbohydrate metabolism and sugar sensing

Not surprisingly, the repression of photosynthesis-related gene expression caused by both treatments was linked with changes in the expression of genes in the metabolism category, with the largest subset of differentially expressed genes belonging to carbohydrate metabolism. Affected genes within this category include those associated with glycolysis, the cleavage of glycosidic bonds, sugar phosphorylation and signal transduction. Thirty-eight carbohydrate metabolism genes showed significantly altered expression in response to NAA, while 149 genes were regulated by shading (Additional file 5, Table S3). NAA-induced genes included those involved with glycolysis and starch degradation, such as pyruvate kinase, alcohol dehydrogenase, amylase and limit dextrinase. Similarly, shading treatment induced genes associated with glycolysis, but also led to changes in the expression of genes for carbohydrate active enzymes, i.e., induction of beta-glycosidases and glycosyltransferase and repression of alpha-glycosidases. Genes related to sucrose metabolism, e.g., sucrose phosphate synthase (*SPS*) and sucrose phosphate phosphatase (*SPP*), were inversely regulated by the two treatments: NAA repressed *SPS *and induced *SPP*, while shading induced *SPS *and repressed *SPP*. A group of genes identified as cytosolic and cell wall invertases were induced by shading in the FAZ, indicating a possible increase in sucrose breakdown in the FAZ. These same invertases were not differentially regulated by NAA, however. The expression of three distinct putative alkaline/neutral invertase genes was reduced after both NAA and shading treatments. Sorbitol dehydrogenase (*SDH*) was repressed by both NAA and shading, while NADP-dependent D-sorbitol-6-phosphate dehydrogenase (*S6PDH*) was induced by NAA but repressed by shading. Many ADP/UDP-glucose pyrophosphorylase genes responsible for starch synthesis were downregulated by shading, but none was differentially regulated by NAA.

Sugar signals are generated from various source organs in response to stresses and changes in metabolic fluxes [25]. Hexokinase (*HXK*) senses glucose levels and SNF-related protein kinases (*SnRKs*) are important to metabolic reprogramming in response to changes in carbohydrate levels [26]. Shading altered the expression of various carbohydrate kinases, but *HXK *was upregulated by NAA only in the FAZ.

Trehalose serves as a storage carbohydrate and stress protectant, which usually accumulates during starvation conditions [25,27]. Trehalose metabolism genes were downregulated by shading but not by NAA treatment. Specifically, genes encoding trehalose-6-phosphate synthase (*TPS*) and trehalose-6-phosphate phosphatase (*TPP*) were repressed in the shading-treated FAZ, suggesting a decreased trehalose level in the FAZ resulted from shading.

### Transport

A large group of transporters for sugars, lipids, amino acids and metal ions were differentially expressed in response to both treatments. The expression of all sorbitol/sucrose transporter genes was consistently repressed by both treatments, while shading downregulated more genes related to general sugar transport, such as hexose transporters. A class of genes related to membrane and cytoskeleton function, including microtubule, vesicle-mediated membrane transporter and cell adhesion genes, were found exclusively repressed by NAA. In all, twice as many transport-related genes were affected by shading compared to NAA, among which several ion transporters, especially for calcium and potassium, were significantly upregulated by shading while others for water transport were downregulated. Another group of transporters, ATP-binding cassette transporters (ABC transporters), were induced by both treatments.

### Cell cycle-related genes

Similar numbers of cell cycle genes were identified in the two datasets of differentially expressed genes, including two classes of regulatory genes, cyclin and cyclin-dependent kinase (*CDK*), being repressed by both NAA and shading. Several cell division control proteins were also downregulated while one CDK inhibitor was upregulated in the FAZ.

### Hormone synthesis and signaling

Many genes involved in different hormone synthesis and signaling pathways showed significant expression changes in response to both treatments. ABA has been implicated as a regulator of stress-induced senescence [28,29]. In this study, NAA appeared to have limited effect on ABA-related genes in that it only upregulated three 9-cis-epoxycarotenoid dioxygenase (*NCED*) genes and a zeaxanthin epoxidase gene, which encode key enzymes in ABA biosynthesis. In contrast, shading altered 26 ABA-related genes involved in biosynthesis, including *NCED*, short chain dehydrogenase/reductase (*SDR*) and abscisic aldehyde oxidase (*AAO*), and several genes related to ABA signaling, including protein phosphatase type 2C and ABA responsive elements-binding factors.

A divergence in auxin-related gene expression was noted for shading- versus NAA-treated trees. Only two auxin-induced SAUR-like and two auxin transport genes showed significant changes in response to shading. However, 21 auxin-related genes were differentially altered by NAA and these genes included IAA-amido synthase, auxin-amidohydrolase, AUX/IAA proteins and various auxin response factors (*ARFs*). Genes related to auxin polar transport were also affected by NAA, with auxin influx carriers induced and efflux carriers largely repressed. The latter effect diverged from that observed for shading-treated trees, where the same auxin efflux carrier genes were induced (Additional file 5, Table S3).

In response to both treatments, genes for ethylene biosynthesis and perception were upregulated, including 1-aminocyclopropane-1-carboxylate synthase (*ACS*) and oxidase (*ACO*) and two classes of ethylene receptors (*ERS *and *ETR*). Coinciding with the increased ethylene biosynthesis (Figure 2), the expression of spermidine synthase gene, a key gene related to polyamine biosynthesis, was consistently reduced by both treatments.

Genes involved with cytokinin and gibberellic acid (GA) signaling pathways were downregulated by shading and NAA. Also, shading increased the expression of a GA2-oxidase gene, which is responsible for GA catabolism. Regarding brassinosteroid (BR)-related genes affected by shading, a BR oxidase gene was repressed, while a BR-signaling kinase gene was induced 3 d after shading. In contrast, expression of BR-related genes was not affected by NAA treatment.

### Cell wall modification

A shared set of 11 genes associated with cell wall biosynthesis, loosening and degradation was responsive to both treatments, with most exhibiting changes at 3 and 5 d after treatment. Specifically, cellulose synthase genes were repressed while other genes related to cell wall loosening and hydrolysis, including β-1,3-glucanase, polygalacturonase and expansin, were all induced.

### Proteolysis and programmed cell death

A number of genes putatively involved in the ubiquitylation pathway were upregulated. Several upregulated genes within the NAA dataset encode F-box proteins and other members of ubiquitin E3 ligase complex, including cullin and ubiquitin-conjugating enzymes. In comparison, shading caused a more widespread induction of genes responsible for protein ubiquitylation and degradation. Shading also had greater impact on the expression of 26S proteasome subunit genes. Another group of genes co-induced by both treatments included those possibly involved in programmed cell death, such as clp and cysteine proteases and autophagy genes. Similar to the pattern observed for cell wall degrading genes, the induction of almost all genes identified in cell death category was detected at 3 and 5 d.

### Transcription factors (TFs)

Several classes of TFs exhibited significant changes in expression. Ten TFs were co-regulated by shading and NAA, including ERF/AP2 transcription factors, bZIP proteins, MADS-box and MYB domain proteins. The differentially expressed ERF/AP2 TFs were co-expressed with the genes for biosynthesis and signaling of ethylene and ABA, consistent with their roles in these two hormone signaling pathways [30,31]. Interestingly, a homolog of the *JOINTLESS *gene (*JNT*), which encodes a MADS-box TF and regulator of abscission zone formation [32], was upregulated by both treatments. While there were both up- and down- regulated NAC domain genes in the shading dataset, *NAC *genes were not differentially expressed in response to NAA. Distinct sets of WRKY TFs were induced by NAA and repressed by shading (Additional file 5, Table S3).

### Validation of array data in the FAZ and analysis of selected genes in other tissues via RT-qPCR

**Figure 4:**
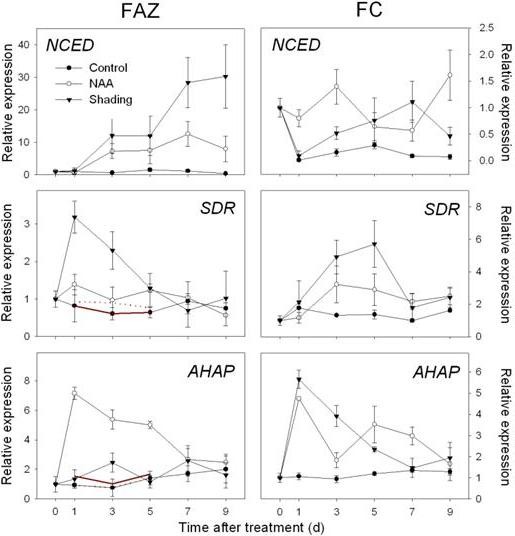
**Expression of genes related to ABA biosynthesis and signaling as determined by RT-qPCR**. Left column, Gene expression in fruit abscission zone (FAZ) from 'Golden Delicious' apple trees after application of NAA and shading. Red lines indicate normalized microarray values (Solid for NAA and dot for shading). Right column, Gene expression in fruit cortex (FC) from 'Golden Delicious' apple trees after application of NAA and shading. The values of transcript levels in the FAZ and FC from control trees were arbitrarily set to 1. The transcript levels were normalized using actin. Results represent the mean (± SE) of three replicates.

**Figure 5:**
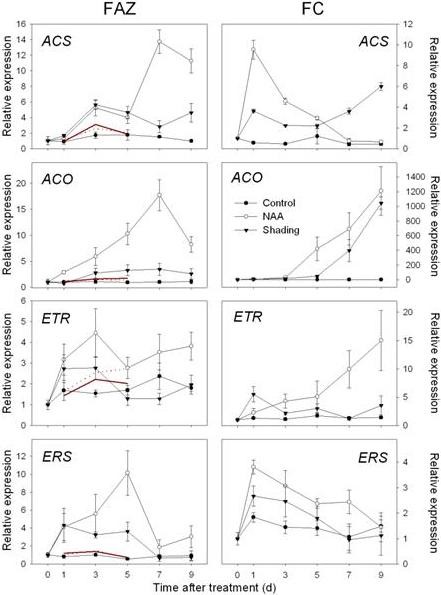
**Expression of genes related to ethylene biosynthesis and signaling as determined by RT-qPCR**. Left column, Gene expression in fruit abscission zone (FAZ) from 'Golden Delicious' apple trees after application of NAA and shading. Red lines indicate normalized microarray values (Solid for NAA and dot for shading). Right column, Gene expression in fruit cortex (FC) from 'Golden Delicious' apple trees after application of NAA and shading. The values of transcript levels in the FAZ and FC from control trees were arbitrarily set to 1. The transcript levels were normalized using actin. Results represent the mean (± SE) of three replicates.

**Figure 6:**
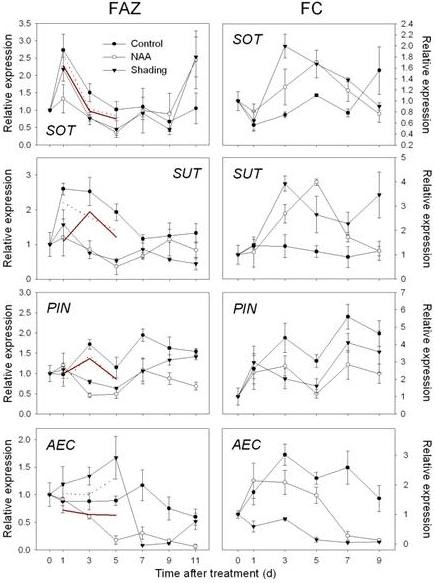
**Expression of genes related to sugar transport and polar auxin transport as determined by RT-qPCR**. Left column, Gene expression in fruit abscission zone (FAZ) from 'Golden Delicious' apple trees after application of NAA and shading. Red lines indicate normalized microarray values (Solid for NAA and dot for shading). Right column, Gene expression in fruit cortex (FC) from 'Golden Delicious' apple trees after application of NAA and shading. The values of transcript levels in the FAZ and FC from control trees were arbitrarily set to 1. The transcript levels were normalized using actin. Results represent the mean (± SE) of three replicates.

**Figure 7:**
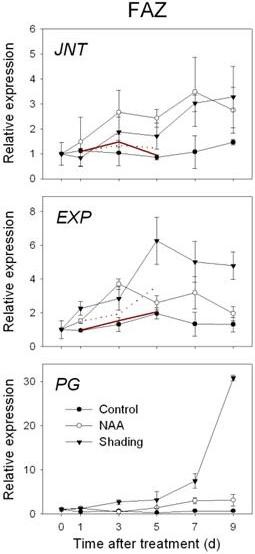
**Expression of genes related to abscission zone formation and cell wall degradation as determined by RT-qPCR**. Gene expression in fruit abscission zone (FAZ) from 'Golden Delicious' apple trees after application of NAA and shading. Red lines indicate normalized microarray values (Solid for NAA and dot for shading). The values of transcript levels in the FAZ from control trees were arbitrarily set to 1. The transcript levels were normalized using actin. Results represent the mean (± SE) of three replicates.

**Figure 8:**
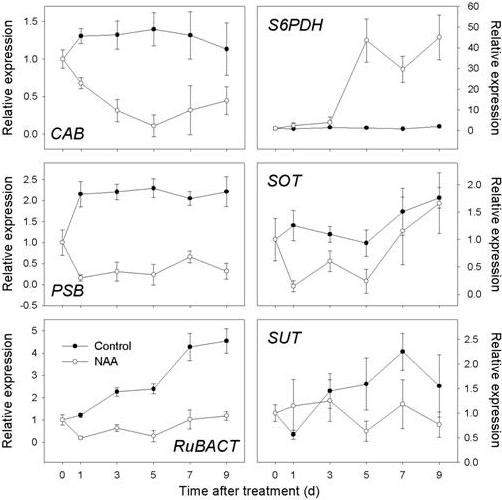
**Expression of genes related to photosynthesis and sugar availability as determined by RT-qPCR**. Gene expression in leaf from 'Golden Delicious' apple trees after application of NAA. The values of transcript levels in the leaf from control trees were arbitrarily set to 1. The transcript levels were normalized using actin. Results represent the mean (± SE) of three replicates.

**Figure 9:**
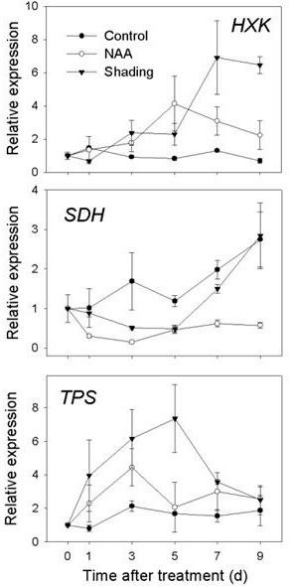
**Expression of genes related to sugar metabolism as determined by RT-qPCR**. Gene expression in fruit cortex (FC) from 'Golden Delicious' apple trees after application of NAA and shading. The value of transcript levels in the FC from control trees were arbitrarily set to 1. The transcript levels were normalized using actin. Results represent the mean (± SE) of three replicates.

Subsets of genes from the above categories were selected for validation of array data in the FAZ by RT-qPCR (Additional files 6 and 7, Table S4 and S5). cDNA samples derived from three additional time points (D0, D7 and D9 after treatment) were included to expand the expression pattern data for these genes. The relative expression levels measured by RT-qPCR were converted to fold change relative to the value obtained from the array data for reference control samples to enable direct comparison to the RT-qPCR results. Generally, the RT-qPCR results from the FAZ samples were consistent with the array data in terms of the overall expression pattern but variations were also observed (Figures 4, 5, 6 and 7). To further explore the effects of NAA and shading on source-to-sink relationships, we analyzed tissue-specific expression pattern of selected genes involved in photosynthesis, sugar metabolism and hormone metabolism and signaling using cDNA derived from leaf and fruit cortex (FC) (Figures 8 and 9).

In both FAZ and FC, the expression of *MdNCED *was induced by both shading and NAA from 3 d. In addition, genes encoding a SDR family protein and a transcription factor (*AHAP*), for the regulation of ABA signaling were induced in the FAZ from 1 to 5 d. In the FC, the expression of those genes was consistently increased by both treatments, especially on 3 and 5 d after treatment (Figure 4). An upregulation of genes encoding ethylene biosynthesis and signaling (*MdACS*, *MdACO*, *MdETR *and *MdERS*) was confirmed by RT-qPCR for NAA- and shading-treated FAZ and mirrored in the FC. Overall, the induction of these ethylene-related genes in the FC was greater in response to NAA than shading, corresponding with the higher levels of ethylene released by fruitlets treated with NAA versus shading (Figure 5 compared to Figure 2). Consistent with the microarray data, both sorbitol and sucrose transporter genes (*MdSOT *and *MdSUT*) were repressed in the FAZ by NAA and shading from 1 to 5 d. In contrast, the expression of these transporters in the FC was increased from 3 through 7 d after both treatments. As for auxin polar transport, a PIN-like auxin transporter gene (*PIN*) and an auxin efflux carrier gene (*AEC*) both showed consistently decreased expression from 3 d in NAA-treated FAZ and FC (Figure 6). Concerning AZ formation and cell wall degradation, the *MdJNT *gene expression in the FAZ was increased by both NAA and shading from 3 d after treatment and remained higher than the control. We also observed an increase of expansin (*EXP*) gene expression in both NAA- and shading-treated FAZ as early as 1 d after treatment, concurrent with the burst of fruit ethylene production (Figure 7 compared to Figure 2). *MdPG2 *expression in the FAZ was induced by NAA and shading from 5 d onward, corresponding with the increased rate of fruit abscission (Figure 7 compared to Figure 1).

Since a widespread repression of chloroplast-related genes in the FAZ was evident from the array data, we further tested leaves to see if photosynthetic organs were similarly affected, RT-qPCR results showed a sustained repression of the selected genes involved with light-harvesting (*CAB*), oxygen evolving enhancement (*PSB*) and Rubisco activation (*RuBACT*) in NAA-treated leaves as early as 1 d after treatment (Figure 8). The expression of genes encoding transporters for both sorbitol and sucrose (*MdSOT *and *MdSUT*) was also found to be repressed in leaves. The expression of three other genes related to sugar metabolism was tested in the FC, which is a site of active carbohydrate metabolism. As shown in Figure 9, *HXK *expression was significantly induced by shading, with maximum levels detected at 7 d. *HXK *expression was most increased by NAA on 5 d, and remained higher than the control level thereafter. The expression of *SDH *gradually increased in the control fruit, but was significantly repressed by NAA and shading. Although our array data showed a consistent downregulation of *TPS *in the FAZ from shading-treated trees, and no effect on *TPS *expression due to NAA, both NAA and shading were shown to cause an early induction of *TPS *in the FC (Figure 9), implicating *TPS *in fruit-specific aspects of abscission independent of the method of induction.

### Effects of NAA on leaf photosynthesis

**Figure 10:**
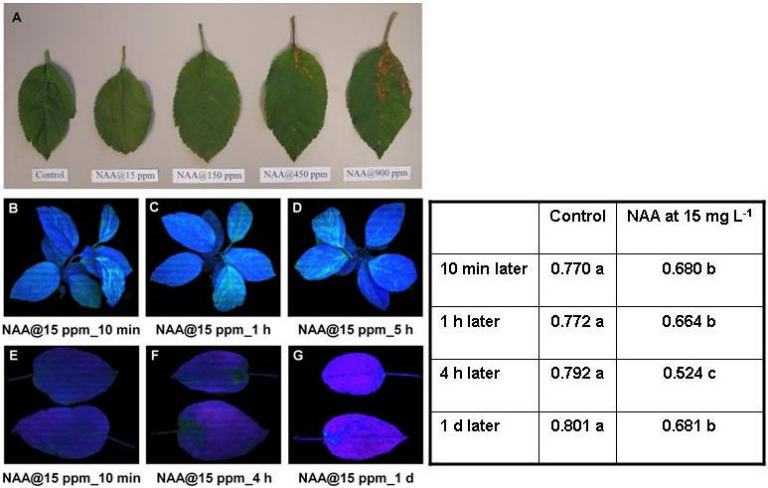
**Effect of NAA at 15 mg L^-1 ^on apple leaf**. (A) White light image of the effect of NAA at various rates on the leaves of young seedlings in growth chamber. Necrosis was observed in a concentration-dependent manner. (B-D) CFI image of the leaves of young seedlings in growth chamber treated with NAA at 15 mg L^-1^. Areas with pale blue color indicate PSII photoinhibition. (E-G) CFI image of the leaves of fruit-bearing trees in the field treated with NAA at 15 mg L^-1^. Areas with dark green color indicate PSII photoinhibition, corresponding with the numerical values (the right column) in the inserted table. Insert table: Effect of NAA at 15 mg L^-1 ^on leaf PSII efficiency. F_V_/F_M _values were recorded three times independently from both control and NAA-affected leaves of fruit-bearing trees in the field. Results represent the mean (± SE) of three replicates. Different letters indicate significant differences among means according to Duncan's multiple range test (*P *≤ 0.05).

From our array data, a large group of genes related to photosynthesis were identified as strongly repressed by NAA in the FAZ at an early stage, implying that NAA might directly interfere with photosynthesis. Therefore, we measured the effect of NAA on the leaf by monitoring the F_V_/F_M _value which provides a useful relative measure of the maximum quantum yield of PSII primary photochemistry. The NAA-affected leaves displayed a unique pattern where the Fv/Fm readings were significantly decreased under fluorescence imaging system, indicating that the leaves were under stress. We also found that NAA at various rates (15, 150, 450 and 900 mg L^-1^) caused concentration-dependent impairment of PSII in the leaves of young seedlings in the growth chamber (Figure 10A). NAA at 15 mg L^-1^, the working concentration used in the thinning experiment, caused significant photoinhibition of leaf PSII efficiency (Figure 10B-D). Such inhibition was observed as early as 10 min post-treatment and lasted for 8 or more hours, from which the leaves typically recovered within 1 d. Next, a field trial on fruit-bearing trees was performed. More severe effects of NAA at 15 mg L^-1 ^on leaf photosynthesis were found and these effects lasted longer than in young seedlings (Figure 10E-G). This increase in severity is not surprising given the higher light levels in the field (full sunlight) versus greenhouse conditions. It is also important to note that under field conditions, the leaves showed visible necrosis by 24 h post-treatment, specifically near the petiole where photoinhibition was most strongly observed. It is not clear if this spatial imbalance was caused by pooling of the NAA solution near the base of the leaf or whether this portion of the leaf is particularly sensitive to NAA. Taken together, these findings indicate that NAA application has rapid and severe impacts on leaf photosynthetic efficiency.

## Discussion

In apple, shading and NAA application are two treatments often applied by researchers to promote fruitlet abscission, while NAA has been widely used by growers to reduce excessive bearing of apple trees. While the transcriptomes associated with abscission in *Arabidopsis *[33-35] and fruit development in apple have been reported [36,37], detailed information on the molecular mechanisms involved in fruit abscission following induction by multiple means remains limited. Using suppression-subtractive hybridization (SSH), various transcripts from shaded small apple fruit have been identified as differentially expressed and over 20% of these are related to carbohydrate metabolism [38]. A recent transcriptome study focusing on the role of benzyladenine (BA) in apple fruit abscission, led to the hypothesis that BA treatment imposes a nutrient stress perceived primarily by the fruitlet cortex and then by the seed, likely through ROS-sugar-ABA signaling, finally leading to abscission zone activation [39]. These findings were specific for the fruit cortex and seed, so did not include information on changes occurring in the FAZ or leaves. It has also been proposed that an increase in ethylene production preceding abscission might hamper the polar auxin transport from the seeds down through the FAZ and cause the fruitlet abscission [40]. The potential importance of auxin-ethylene crosstalk was also partly supported by a tomato flower AZ transcriptome analysis [17], where auxin depletion caused altered expression of auxin-regulated genes in association with the acquisition of ethylene sensitivity in the AZ. While these findings implicate auxin-ethylene crosstalk in abscission, they did not focus on plant stress response linked with metabolic reprogramming as a factor that influences hormone signalling for abscission. In addition, these studies have not examined whether distinct treatments for inducing abscission, such as shading and NAA, converge on common genetic pathways leading to abscission.

In this study, by comparing the gene expression profiles of young fruit abscission caused by NAA and shading, we found that the number of genes affected by these two treatments was positively correlated with the severity of the thinning responses. Our microarray experiments involved sampling during induction of abscission and the beginning of a detectable increase in the rate of abscission rather than during the abscission process itself. Hence, additional work is required to determine changes in gene expression occurring during later stages of abscission. Although any large changes in expression limited to narrow windows of either temporal or spatial regulation in the abscission zone were likely attenuated due to sampling that included mixed fruitlets of different abscission potentials, the changes in gene expression we observed confirm earlier reports of the involvement of two important hormone signaling pathways, ethylene and ABA [12,13]. Moreover, our array data showed that NAA may exert its thinning effect through interfering with leaf photosynthesis, as well as sugar sensing and carbohydrate partitioning within the tree, which is similar to pathways involved in the shading-induced young fruit abscission in apple.

### Hormone regulation in fruit abscission

Our microarray results are consistent with previous observations that ethylene is a key signal for the coordination of young fruit abscission induced by the chemical thinner NAA in apple [12,13,41]. In this study, ethylene production increased and peaked in fruitlets and leaves treated by NAA and shading, coincident with upregulation of genes encoding ethylene biosynthesis and signaling components but prior to the onset of fruitlet drop (Figures 1, 2 and 5). These results were consistent with a previous report that apple fruitlet abscission is preceded by an increase in ethylene biosynthesis and sensitivity [12], although ethylene independent pathways may also promote fruit abscission [41].

It has also been hypothesized that preventing abscission requires a constant auxin transport through the abscission zone from the fruit and that the sink strength of organs is related to their capacity to produce and export auxin [42,43]. Auxin export is mediated by PIN-formed proteins and ATP-activated phosphoglycoproteins (*PGPs*) [44,45]. We found that two auxin transport-related genes (*PIN *and *AEC*) were repressed by NAA in the FAZ and FC, i.e., starting from 3 d, and their expression levels remained lower than the control, indicating that auxin efflux from the fruitlets was blocked (Figure 6). Auxin transporter downregulation was associated with increases in ethylene production and the expression of ethylene biosynthesis and signaling-related genes (Figures 5 and 6). Using the tomato flower AZ model, Meir and colleagues [17] noted that the acquisition of ethylene sensitivity was associated with altered expression of auxin-regulated genes and that ethylene acted as a trigger in the abscission process, although these authors did not measure the expression of *PIN *genes. Taken together, the patterns of ethylene- and auxin-related gene expression in the FAZ and FC suggest that ethylene may serve as a feedback inhibitor controlling auxin transport from fruitlets, and increasing the ethylene sensitivity of the FAZ.

Abscisic acid has been implicated in the regulation of stress-induced senescence [29], and it has been proposed that ABA might sense nutrient stress and be correlated with the ethylene-associated abscission activation in citrus fruitlets [46]. We observed a widespread induction of genes involved with ABA biosynthesis and signalling in response to shading and an increase in the expression of genes for ABA biosynthesis with NAA treatment (Additional file 5, Table S3, Figure 4). These differences in ABA-related responses suggest that the ABA signaling pathway is responsive to both treatments, but is more active in shading-induced fruit abscission. As mentioned above, more genes associated with sugar metabolism and sugar signaling were altered by shading than NAA, implicating a close crosstalk between sugar and ABA for the induction of senescence as previously reported [47].

### Repression of photosynthesis-related genes

Decreased light intensity can inhibit photosynthesis and result in the abscission of leaves and fruitlets [7], which was confirmed by our shading experiment. We also found that NAA caused observable leaf necrosis and diminution of overall PSII efficiency (Figure 10), which agreed with a previous report [48], where NAA consistently reduced whole-tree canopy photosynthesis. Repression of a group of chloroplast-related genes was also observed for NAA-treated leaves (Figure 8), and these gene expression patterns also agreed with the array data for FAZs from NAA-treated trees, indicating that NAA affected photosynthesis in both leaf and stem tissues. The negative effect of NAA on leaf photosynthesis and PSII activity implicates NAA in causing a carbohydrate stress that ultimately affected sink tissues, including the fruitlets.

### Impacts on sugar metabolism, sensing and transport

It has been reported that dark-induced fruit abscission can be reversed with trunk injection of sorbitol, the primary translocated form of carbohydrate in apple [49], which supports the currently accepted hypothesis that a limitation of assimilate supply at least partly reduces fruit growth and induces fruit drop. Plant hexokinase has been implicated in sugar signaling and the regulation of senescence [50,51]. As a kinase and glucose sensor, the *HXK *gene has been demonstrated to have dual functions in both glucose metabolism and signaling [50,51]. In this study, we found that a homolog of the *HXK *gene in the FC was induced by both shading and NAA treatments (Figure 9), suggesting a HXK-dependent sugar-signaling pathway is active during the abscission induction. It has been reported that transgenic tomato plants that overexpress *Arabidopsis HEXOKINASE1 *showed inhibited growth and rapid senescence [52], while *Arabidopsis glucose insensitive2 *(*gin2*) mutant plants displayed a delayed senescence phenotype [51]. Thus the elevated expression level of *HXK *observed in this study might partly account for the inhibited fruit growth and accelerated fruit abscission.

Sorbitol comprises over 80% of the carbohydrate translocated in the phloem of apple, and thus is the main carbon resource imported by fruit sinks [49]. Sorbitol dehydrogenase has been identified as a key enzyme in sorbitol metabolism, converting sorbitol into fructose [53,54]. In this study, the expression of *SDH *gradually increased in the control fruit, indicating its role in regulating early fruit development. However, NAA and shading both repressed the expression of *SDH *in the FC (Figure 9), suggesting that sorbitol catabolism was largely inhibited and fruit sink strength was impaired [55], resulting in abscission. Another important sorbitol-metabolizing enzyme is sorbitol-6-phosphate dehydrogenase (*S6PDH*) that synthesizes sorbitol in leaves for the translocation to sink tissues [56]. Previous studies have reported a relation between an increase in soluble carbohydrates and stress tolerance in some Rosaceae fruit trees [57,58], and *S6PDH *has been revealed as an ABA-inducible gene [59]. We observed a strong induction of *S6PDH *expression in NAA-treated leaves (Figure 8), and the induction of genes related to ABA biosynthesis and signaling in both FAZ and FC preceded the increased *S6PDH *expression in leaf (Figure 4), consistent with *S6PDH *being an ABA-mediated stress response gene.

NAA and shading both significantly inhibited certain sugar transporter genes in the FAZ and leaves, indicating that the carbon allocation to the fruitlets was hampered. However, the expression of these transporters in the FC was increased after both treatments (Figure 6). Also, the array data revealed that many transporter genes were induced by NAA, including a number of ABC transporters and several cation transporters, which was consistent with previous findings [33]. Also induced in this study were several nitrate and sulfate transporter genes. The upregulation of these transporters, especially those involved in sugar transport, might reflect the shifting function of abscising fruitlets, as they become a source tissue for the mobilization of nutrients to non-abscising fruitlets.

### Induction of genes associated with FAZ formation and cell wall degradation

The cells comprising the AZ are often morphologically distinguishable before the onset of abscission [60] and abscission could not be induced until those cells are formed [61]. The tomato *JOINTLESS *gene is a MADS-box gene that plays a key role in controlling abscission zone development [32]. In this study, the *MdJNT *expression in the FAZ was gradually increased by both NAA and shading, suggesting that this transcription factor involvement in abscission zone formation in apple and tomato is conserved.

Enlargement of abscission zone cells involves cell wall loosening. Wall loosening can be aided by expansins and several reports have shown that expansins are expressed abundantly in abscission zones [22,62]. We also observed an increase in expansin (*EXP*) expression in both NAA- and shading-treated FAZ as early as 1 d after treatment, concurring with the burst of fruit ethylene production. It has been reported that certain types of AZ cells enlarge in response to ethylene [61]. Together these results support a role of expansins in cell enlargement associated with ethylene-mediated abscission.

Some reports have indicated that an increase in polygalacturonase (*PG*) activity correlates with fruit abscission [18,63]. However, *MdPG1 *expression was not detected in the FAZ or the FC from 'Golden Delicious' or 'Delicious' apples [13], which contrasts with other work showing that *MdPG1 *was involved in apple fruit softening and that its expression was suppressed by 1-MCP and AVG treatment [16,64]. In this study, *MdPG1 *was not identified from the array but the expression of *MdPG2 *in the FAZ was induced by NAA and shading by 5 d, occurring with the increase in the rate of fruitlet abscission. This result was in agreement with our previous work with NAA in 'Delicious' apple [13]. Another report showing an *MdPG2 *downregulation concomitant with an NAA-dependent reduction in preharvest fruit drop [16] supports our current view that *MdPG2 *rather than *MdPG1 *appears to be strongly associated with fruitlet abscission.

## Conclusions

**Figure 11:**
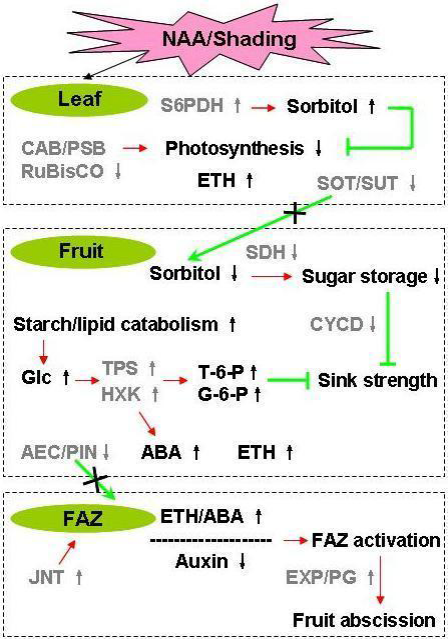
**A hypothetical model for NAA-induced young fruit abscission in apple**. NAA, like shading, imposes a stress signal on leaf, or globally on any other photosynthetically-active tissues within the tree, causing photosynthesis repression and associated nutrient stress. As the nutrient stress is perceived at the fruit level, its growth is inhibited by a sugar transport block, resulting in a lower sink strength of the fruitlet. Meanwhile, ethylene and/or ABA are produced in response to photosynthesis inhibition and through sugar signaling. The elevated ethylene level decreases auxin transport to the FAZ and increases its sensitivity to ethylene, causing the differentiation of the FAZ and the execution of fruit abscission.

The objective of this study was to compare global gene expression changes at early stages during apple fruitlet abscission caused by two different abscission inducers: the chemical thinner NAA and shading. A model has been proposed here to illustrate the association of gene expression changes in common with both abscission inducers during the early induction of fruitlet abscission (Figure 11). In summary, NAA, like shading, imposes a stress signal on leaf, or globally on any other photosynthetically-active tissues within the tree, causing photosynthesis repression and associated nutrient stress. As the nutrient stress is perceived at the fruit level, its growth is inhibited by a sugar transport block, resulting in a lower sink strength of the fruitlet. Meanwhile, ethylene and/or ABA are produced in response to photosynthesis inhibition and through sugar signaling. The elevated ethylene level decreases auxin transport to the FAZ and increases its sensitivity to ethylene, causing the differentiation of the FAZ and the execution of fruit abscission. The differential gene expression data presented in this study allows for the development of novel hypotheses regarding genes that are important regulators of fruit abscission. These hypotheses can be functionally tested, using RNA interference or virus-induced gene silencing, with genes identified through the recent release of the apple genome [65]. Moreover, the results of this study may facilitate the selection of new chemicals or genetic strategies for the development of more effective apple fruit thinning programs.

## Methods

### Plant materials and sample collection

Thirty-six uniform 12-year-old 'Golden Delicious' apple trees on M.9 rootstock were selected and divided into three blocks of 12 trees each. Four trees from each block were treated with: 1) Control (water); 2) NAA (Fruitone N; AMVAC, Newport Beach, CA) at 15 mg L^-1^; 3) Shading (92% polypropylene shade over the entire tree for five consecutive days and then removed), respectively. Treatments were applied when the fruit size was ~10 mm in diameter. Three biological replicates were conducted independently. Young fruit was collected at 0, 1, 3, 5, 7, 9 d after treatment. At each collection time, about 80 fruit were collected from each tree, with fruit cortex (FC) and fruit abscission zone (FAZ) separated. FAZs were collected by cutting 1 mm at each side of the abscission fracture plane at the base of the pedicel [41]. All samples were promptly frozen in liquid nitrogen and stored at -80°C for future RNA extraction.

### Fruit abscission pattern and leaf/fruit ethylene production

To determine the fruit abscission rate, two limbs on each tree were tagged and fruit on tagged limbs were counted on 0, 1, 2, 3, 7, 9, 11, 14, 16, 18, 21, 25 and 26 d after treatment. For ethylene production measurements, 15 fruit and 20 leaves were collected from each of three replicate trees 0 and 6 h and 1, 3, 5, 7, 9, 11 and 14 d after treatment and enclosed in a 100-mL (for fruit) or 1000-mL (for leaves) container. After a 2-h incubation period, a 1-mL gas sample was withdrawn from the sealed container through the rubber septum affixed to the lid, and the ethylene concentration was measured with a gas chromatograph equipped with an activated alumina column and FID detector (model 3700; Varian, Palo Alto, CA) and expressed as μL C_2_H_4 _kg^-1 ^h^-1^.

### Experimental design and microarray hybridization

An apple 70-mer oligonucleotide microarray consisting of ~33,825 unique sequences and ~ 6,000 controls was used [66]. RNA from three time points (D1, 3 and 5) were represented by three biological replicates analyzed in a dye swap design (six hybridizations per time point) for a total of 18 slides for the NAA treatment and for the shading treatment. A control RNA (the untreated samples of the same time point) was hybridized along with the treatment RNA but with the opposite dye. A total of 50 pmol of incorporated dye with at least a FOI of 2.0 (calculated using Base/Dye Ratio Calculator from Invitrogen) was used for each sample cDNA and the reference cDNA in the hybridization.

### RNA extraction, aRNA amplification and labeling

Total RNA was extracted from the FAZ for each biological replicate as previously described [16], and DNased using TURBO DNA-free™ Kit (Ambion, Austin, TX). RT-PCR was performed using primers that span an intron in *MdACO *to confirm that each RNA sample was free of genomic DNA contamination. The RNA was quantified using the NanoDrop ND-1000 (Thermo Scientific, MA) and the quality checked using the Bioanalyzer 2100 (Agilent, CA) according to manufacturer's instructions. According to the Instruction Manual of Amino Allyl MessageAmp™ II aRNA Amplification Kit, cDNA was synthesized from mRNA in 1 μg of total RNA. Purified cDNA was transcribed to aRNA using IVT Master Mix which contains 5-(3-aminoallyl)-UTP (50 mM), ATP/CTP/GTP Mix (25 mM), UTP (50 mM), T7 10 × Reaction Buffer and T7 Enzyme Mix. Purified aRNA was labeled with either AlexaFluor555 or AlexaFluor647 (Invitrogen, CA) for hybridization following the manufacturer's instructions. Labeled samples (10 μL) were mixed with 1 × Slide Hyb Glass Hybridization Buffer (Ambion) and injected into the slide chambers which were heated to 65°C. The chambers were incubated at 42°C overnight. The next day, the slides were washed with 1 × SSC, 0.2% SDS for 5 min, 0.1 × SSC, 0.2% SDS for 5 min and twice with 0.1 × SSC.

### Data scanning and analysis

Microarray slides were dried and dual channel images were captured using GenePix 4000B microarray scanner (Axon Instruments, CA). Automated spot alignment was augmented with manual checking of each slide to remove substandard spots. GenePix Pro software (Axon Instruments, CA) was used for data normalization and statistical analysis. LIMMA package for R programming environment was used by applying linear model methods [67]. Each probe was tested for changes in expression over the time points using a moderated F test, which is similar to an ANOVA method for each probe except that the residual standard errors are moderated across genes [68]. The linear models allow for general changes in gene expression between successive time points. The use of dye-swaps in the experimental design eliminated a dye-effect for each probe, which increased the precision with which differential expression could be detected. The computed *P *values were adjusted for multiple testing to control the false discovery rate (FDR) [69]. Genes were considered significantly expressed if the adjusted *P *values were <0.01 (i.e. expected FDR less than 1%).

### Gene clustering and categorization

Hierarchical clustering was performed using the statistic package for R utilizing the Euclidean distance. Figure of merit (FOM) analysis was performed to determine the number of clusters needed for the explanation of the majority of variation in expression patterns [70]. Then a cluster number was assigned for K-means clustering (KMC) analysis to divide the data into distinct expression clusters based on similarities in their expression patterns, using the TM4 package [70]. Default statistical parameters were used in those analyses and data were scaled for hierarchical and KMC clustering based on fold-change and log_2 _ratio in gene expression. The existing apple gene annotation was complemented by the application Blast2Go [71] and further supplemented with manual BLASTX, conserved domains and literature searches, mostly based on *Arabidopsis *database. Using this combined information, a functionally driven classification was created manually. Larger categories were further divided into subcategories to cover all the related genes.

### RT-qPCR

Purified total RNA (1 μg) from each sample was used to synthesize cDNA in a 20-μL reaction using the High-Capacity cDNA Reverse Transcription kit (Applied Biosystems, Fosters City, CA). Each qPCR reaction was run in triplicate using 40 ng of cDNA in a 15-μl reaction, using Power SYBR Green qRT-PCR Kit (Applied Biosystems). Gene-specific primer sets were designed from available apple ESTs sequences using Primer Expression 3.0 and synthesized by Integrated DNA Technologies (Coralville, IA) [13]. Upon the release of the apple genome [65], we checked if our qPCR primers were specific to a single gene or could potentially amplify multiple gene family members using BlastN searches. Those that could amplify more than one gene were discarded (Additional file 7, Table S5). The reactions were performed on a 7500 Real-time PCR Cycler (Applied Biosystems, CA). Quantification was achieved using a relative standard curve derived from a standard RNA run in parallel with each primer set. A primer set designed to amplify *Malus *actin RNA was run on all samples and used to normalize the data. A dissociation curve was run to verify that a single desired amplified product was obtained from each reaction (Applied Biosystems).

### Measurement of leaf photosynthesis efficiency

NAA was directly applied to the leaves of both young apple seedlings in a growth chamber (21-24°C, 50% humidity, light-dark cycle of 16:8 h; the seedlings were treated 4 h after the light cycle began) and mature fruit-bearing apple trees in the field (4 h after sunrise, full sunlight). After treatment, the young seedlings and separate leaves from the mature trees were collected at intervals (10 min, 1 h and 5 h for young seedlings; 10 min, 4 h and 1 d for mature trees). Chlorophyll fluorescence images (CFI) were taken and Fv/Fm values were recorded to monitor the changes in PSII efficiency, using an IMAGING-PAM Fluorometer with Walz ImagingWin software V2.32. F_V_/F_M _values were recorded three times independently from both control and NAA-affected leaves of fruit-bearing trees in the field.

## Authors' contributions

RY conceived the project, acquired the funding and designed the experiment. HZ participated in the experimental design, carried out chemical treatments and array experiment, conducted data analyses and prepared the manuscript. CDD participated in the experimental design, oversaw the data analyses and edited the manuscript. EPB and AMC participated in the results discussions and provided extensive intellectual suggestion for the manuscript organization and writing. RX contributed to the experimental design and array data analyses. All authors critically read and approved the final version of the manuscript.

## Supplementary Material

Additional file 1 **Supplementary Table S1. Table S1 - Statistically significant genes derived from apple fruit abscission zone microarray study**. Click here for file

Additional file 2 **Supplementary Figure S1. Figure S1 - Hierarchical cluster of 722 selected genes from NAA-treated FAZ (A) and 1057 selected genes from shading-treated FAZ (B) from the 40 K apple microarray**. The fold changes in gene expression are scaled from 0.5 to 2.0 to allow clustering by expression pattern, with intense red representing maximum expression and intense green representing minimum expression. Click here for file

Additional file 3 **Supplementary Table S2. Table S2 - K-means clustering for NAA- and shading-treated apple fruit abscission zone microarray data**. Click here for file

Additional file 4 **Supplementary Figure S2. Figure S2 - Clusters of NAA-responsive genes (A) and shading-responsive genes (B) with average values (pink line) and standard deviation (grey area) of the expression levels of the selected genes are presented**. In these diagrams, "y" axis represents log_2_-fold change and "x" axis represents the different time points for sampling. The cluster names are assigned upregulated (u), unchanged (o) or downregulated (d) for each time point. Click here for file

Additional file 5 **Supplementary Table S3. Table S3 - Categorization of significant genes encoding enzymes with a variety of biological functions**. In this table, eight functional categories of genes showing differential expression patterns after NAA and shading treatments, from the array data are presented. A comparative heat map is also included. The fold change scale is shown at top along with the time points and gene categories are listed along with the color bars. Click here for file

Additional file 6 **Supplementary Table S4. Table S4 - Summary of array-measured expression of genes modified at early stages (D1, 3 and 5) after NAA and shading treatments**. '+' and '-' signs represent up- and down-regulation of genes, respectively, while 0 represents no change. Click here for file

Additional file 7 **Supplementary Table S5. Table S5 - Real-time qPCR primers**. A list of primer sequences and gene accession numbers used for quantitative polymerase chain reaction studies. Click here for file
