# Supplementary material for: Transcriptomics of shading-induced and NAA-induced abscission in apple (Malus domestica) reveals a shared pathway involving reduced photosynthesis, alterations in carbohydrate transport and signaling and hormone crosstalk
Source: BMC Plant Biol. 2011 Oct 17;11:138. doi: 10.1186/1471-2229-11-138 (PMC3217944; doi:10.1186/1471-2229-11-138)
Supplement: Additional file 2 — Supplementary Figure S1. Figure S1 - Hierarchical cluster of 722 selected genes from NAA-treated FAZ (A) and 1057 selected genes from shading-treated FAZ (B) from the 40 K apple microarray. The fold changes in gene expression are scaled from 0.5 to 2.0 to allow clustering by expression pattern, with intense red representing maximum expression and intense green representing minimum expression. [file 1471-2229-11-138-S2.PDF]

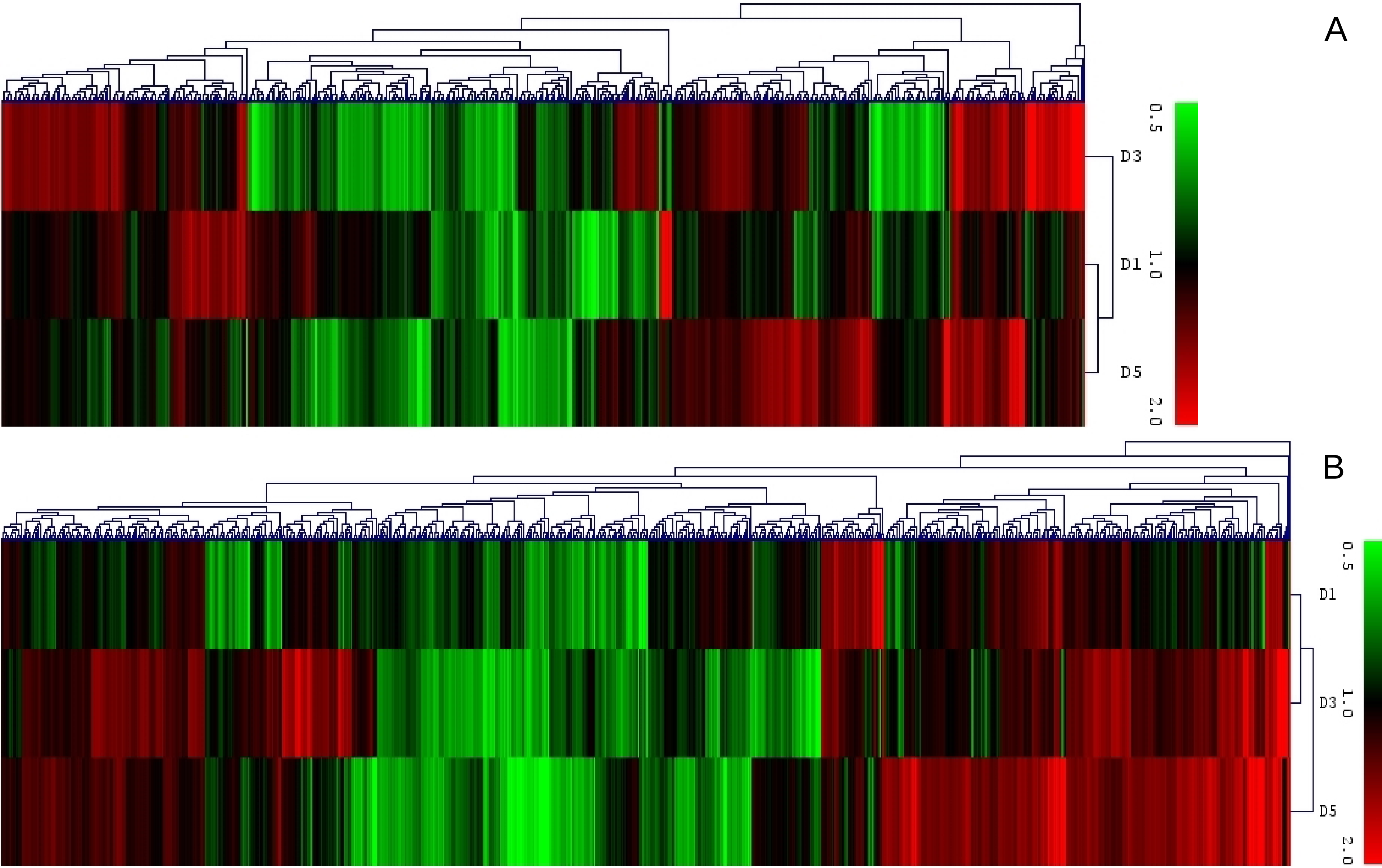

Additional file 2 – Hierarchical cluster of 722 selected genes from NAA-treated FAZ (A) and 1057 selected genes from shading-treated FAZ (B) from the 40 K apple microarray. The fold changes in gene expression are scaled from 0.5 to 2.0 to allow clustering by expression pattern, with intense red representing maximum expression and intense green representing minimum expression.
