# Supplementary material for: Transcriptomics of shading-induced and NAA-induced abscission in apple (Malus domestica) reveals a shared pathway involving reduced photosynthesis, alterations in carbohydrate transport and signaling and hormone crosstalk
Source: BMC Plant Biol. 2011 Oct 17;11:138. doi: 10.1186/1471-2229-11-138 (PMC3217944; doi:10.1186/1471-2229-11-138)
Supplement: Additional file 4 — Supplementary Figure S2. Figure S2 - Clusters of NAA-responsive genes (A) and shading-responsive genes (B) with average values (pink line) and standard deviation (grey area) of the expression levels of the selected genes are presented. In these diagrams, "y" axis represents log2-fold change and "x" axis represents the different time points for sampling. The cluster names are assigned upregulated (u), unchanged (o) or downregulated (d) for each time point. [file 1471-2229-11-138-S4.PDF]

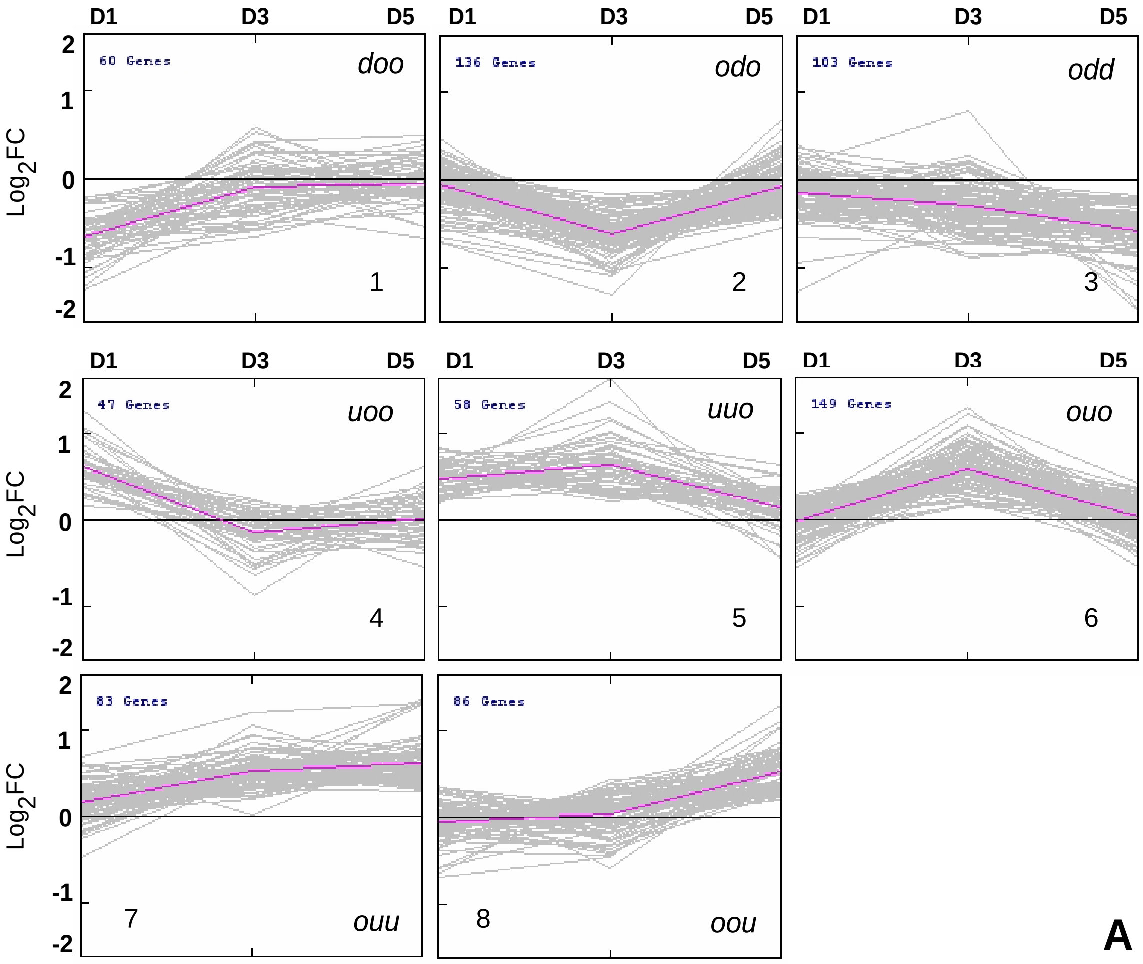

Additional file 4 – Clusters of NAA-responsive genes with average values (pink line) and standard deviation (grey area) of the expression levels of the selected genes are presented. In these diagrams, “y” axis represents log_2-fold change and “x” axis represents the different time points for sampling. The cluster names are assigned upregulated (u), unchanged (o) or downregulated (d) for each time point.

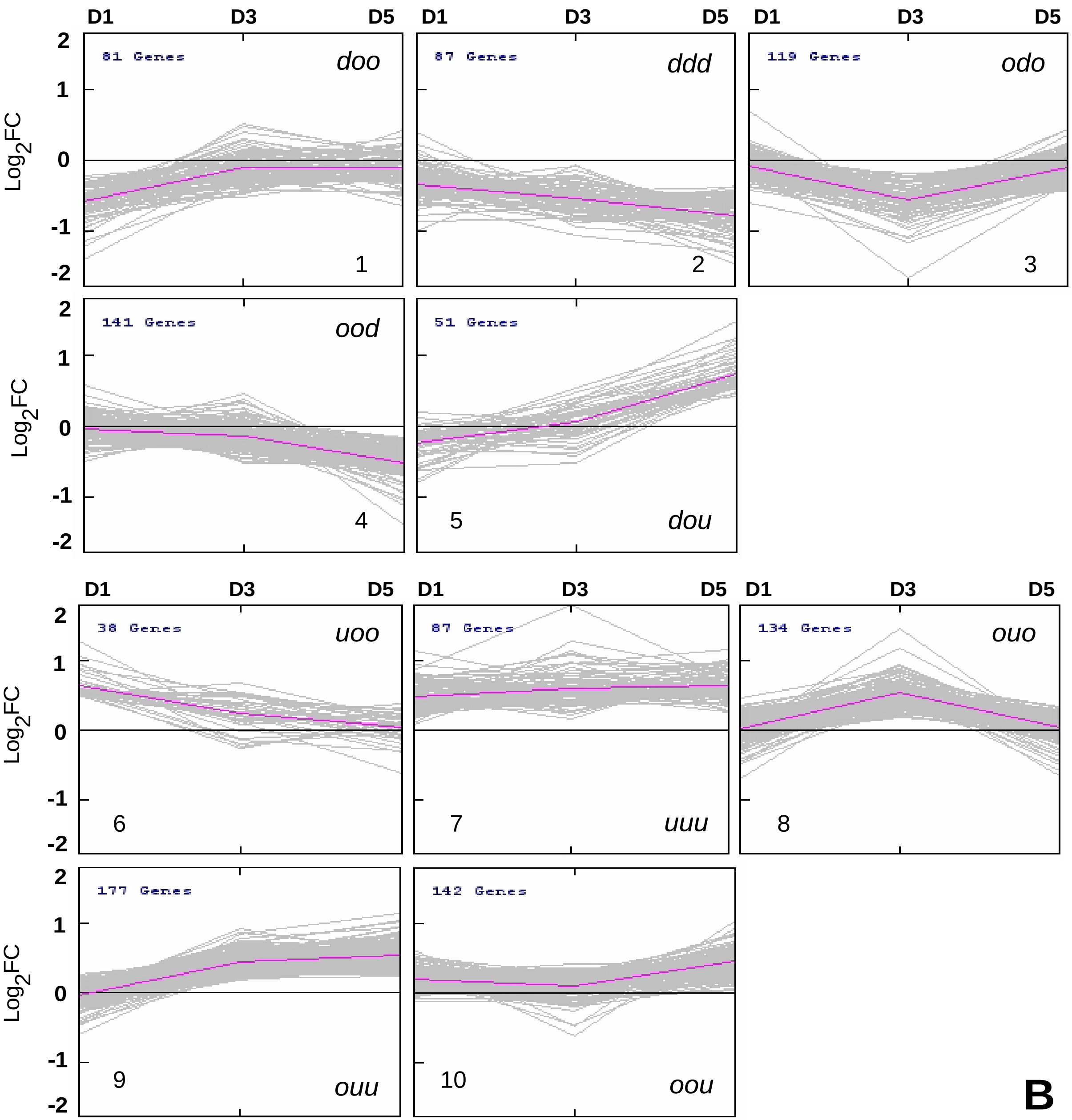

Additional file 4 – Clusters of shading-responsive genes with average values (pink line) and standard deviation (grey area) of the expression levels of the selected genes are presented. In these diagrams, “y” axis represents log_2-fold change and “x” axis represents the different time points for sampling. The cluster names are assigned upregulated (u), unchanged (o) or downregulated (d) for each time point.
